# Molecular stool testing as an alternative for surveillance colonoscopy: a cross-sectional cohort study

**DOI:** 10.1186/s12885-017-3078-y

**Published:** 2017-02-07

**Authors:** Meta C. J. van Lanschot, Beatriz Carvalho, Veerle M. H. Coupé, Manon van Engeland, Evelien Dekker, Gerrit A. Meijer

**Affiliations:** 1 Department of Pathology, The Netherlands Cancer Institute, Plesmanlaan 121 1066 CX, Amsterdam, The Netherlands; 2 Department of Gastroenterology and Hepatology, Academic Medical Center, Meibergdreef 9 1105 AZ, Amsterdam, The Netherlands; 3 Department of Epidemiology and Biostatistics, VU medical center, De Boelelaan 1118, 1081 HZ Amsterdam, The Netherlands; 4 GROW-School for Oncology and Developmental Biology, Maastricht University Medical Center, Universiteitssingel 40 6229 ER, Maastricht, The Netherlands

**Keywords:** Polypectomy, Colorectal cancer, Surveillance, Multitarget stool DNA testing, Cologuard®, FIT

## Abstract

**Background:**

As in many other European countries, a nationwide screening program for colorectal cancer (CRC) has recently been introduced in the Netherlands. As a side effect, such a screening program will inherently yield an increase in the demand for surveillance after removal of polyps/adenomas or CRC. Although these patients are at increased risk of metachronous colorectal neoplasia, solid evidence on CRC-related mortality reduction as a result of colonoscopy-based surveillance programs is lacking. Furthermore, colonoscopy-based surveillance leads to high patient burden, high logistic demands and high costs. Therefore, new surveillance strategies are needed. The aim of the present study, named Molecular stool testing for Colorectal CAncer Surveillance (MOCCAS), is to determine the performance characteristics of two established non-invasive tests, i.e., the multitarget stool DNA test Cologuard® and the faecal immunochemical test (FIT) in the detection of CRC and advanced adenomas as an alternative for colonoscopy surveillance.

**Methods:**

In this observational cross-sectional cohort study, subjects aged 50 to 75 years will be approached to collect (whole-) stool samples for molecular testing and a FIT prior to their scheduled surveillance colonoscopy. The results of the tests will allow calculation of test sensitivities and specificities in the context of surveillance. This will provide the required input for the Dutch ASCCA model (Adenoma and Serrated pathway to Colorectal CAncer) to simulate surveillance strategies differing in frequency and duration. The model will allow predictions of lifetime health effects and costs. Multiple centres in the Netherlands will participate in the study that aims to include 4,000 individuals.

**Discussion:**

The outcome of this study will inform on the (cost-) effectiveness of stool based molecular testing as an alternative for colonoscopy in the rapidly expanding surveillance population.

**Trial registration:**

ClinicalTrials.gov (https://clinicaltrials.gov/): NCT02715141. Retrospectively registered 17 February 2016.

## Background

Colorectal cancer (CRC) is a major health concern worldwide, ranking third in males and second in females, with over 1.2 million new cancer cases and an estimated 608,700 deaths in 2008 [1]. Survival of colorectal cancer is inversely related to the stage at diagnosis. Five-year survival rates range from more than 90% for stage I to less than 10% for stage IV CRC [2]. Therefore, detection and removal of the tumour in an early, or preferably, a premalignant stage is vital [3]. Since CRC often only becomes symptomatic when progressed to an advanced state, secondary prevention through screening is an important instrument for reducing death from CRC [4].

CRC as a disease lends itself well for screening as it has a high prevalence and a well-defined precursor lesion (i.e., adenoma) with a long dwell-time, providing an excellent window of opportunity for detection and resection of a lesion before becoming symptomatic. Indeed, incidence and mortality rates of CRC have declined in countries where screening has been introduced [5–7]. Recently a nationwide screening programme was also implemented in the Netherlands, using a faecal immunochemical test (FIT). Men and women aged 55 to 75 years are invited every 2 years, which amounts to a total number of 2.2 million individuals per year in the Netherlands being invited to participate in the program. Of the estimated 7% FIT-positive screenees, approximately half will have advanced adenomas or carcinomas at colonoscopy, equal to over 45.000 individuals annually [8]. As these patients carry an increased risk to develop metachronous advanced lesions in the future [9–12] it is standard practice to enrol these individuals in a colonoscopy-based surveillance program [13–15].

There are several downsides, though, to the current approach for managing the cancer risk in the surveillance population. Firstly, evidence of the impact of current surveillance strategies on the ultimate endpoint CRC-related mortality is very limited. The effect of surveillance has primarily been evaluated for intermediate endpoints, i.e., the yield of (advanced) adenomas upon surveillance colonoscopy [12, 16]. However, adenomas are very common with a prevalence of 18-35% reported in screening series [17, 18], whilst only up to 5% of these adenomas will eventually progress to malignancy [19]. This suggests that focussing on adenoma yield as a primary endpoint represents actual overdiagnosis. Moreover, the technical advancements in colonoscopy-equipment and the recent emphasis on quality assurance have resulted in increasingly more and often smaller adenomas being detected. These small lesions are more likely to remain stable over time [20, 21]. As a result, the surveillance population will expand even further, putting the colonoscopy capacity and health care budgets under pressure [22, 23]. Finally, it will expose post-polypectomy patients to a burdensome and risky procedure that has not proven to be effective for this population.

For these reasons there is a demand for a surveillance tool that is easy to apply, well-tolerated and accurate in identifying high risk adenomas, as to reserve colonoscopy only for those individuals that are most likely to benefit.

Today, despite its limitations, colonoscopy is still the only test used for surveillance of patients after removal of polyps and CRC. The FIT, which detects small amounts of human haemoglobin in the faeces, has been proposed as a method for surveillance [24]. While simple and increasing the likelihood of neoplasia being present when positive, its sensitivity is relatively low due to the fact that not all CRCs, and especially not all advanced adenomas bleed [25, 26]. Yet, it was shown that the diagnosis of CRC and advanced adenomas was made 25 and 24 months (median) earlier, respectively, when offering a yearly FIT in the interval between colonoscopies and shortening the interval in case of a positive test. This indicates that FIT could be used to detect missed or rapidly developing lesions in surveillance programs [24].

In contrast to tests detecting blood, tests based on molecular markers derived from the neoplastic cells in the colon have the potential to be more accurate. CRCs are known to acquire discriminating epigenetic and genetic changes as they develop and progress, which form the basis of stool based DNA testing. Recently, a multitarget stool DNA test (Cologuard®, Exact Sciences, Madison, WI, USA) combining a molecular assay for hypermethylated promoter CpG islands (*NDRG4* and *BMP3*) and mutant *KRAS* with an immunoassay for human haemoglobine has been reported on [27]. Sensitivities for the detection of CRC were 92.3% with Cologuard® and 73.8% with FIT (p =0.015). Also, Cologuard® detected significantly more advanced adenomas than the FIT (69.2% versus 46.2%, p = 0.004) and significantly more sessile serrated polyps measuring 1 cm or more (42.4% versus 5.1%). Specificities of Cologuard® and FIT were 86.6 and 94.9%, respectively. The results of this study have led to FDA approval of Cologuard® in 2014.

We hypothesise that Cologuard®- or FIT-based surveillance is a cost-effective first-line surveillance strategy to select individuals that need colonoscopy for diagnosis confirmation and therapeutic removal.

## Objective

### Primary objective

**Fig. 1:**
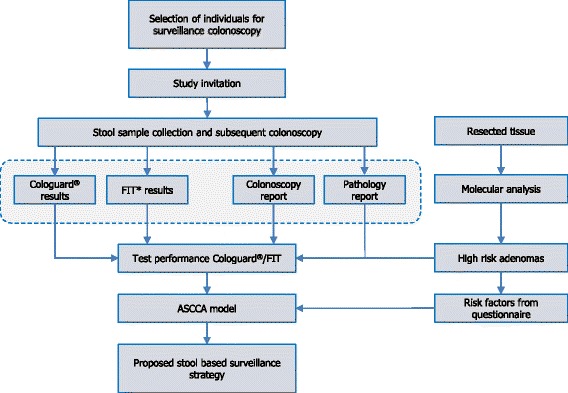
Overview of the MOCCAS study. * FIT = faecal immunochemical test, consisting of the OC-sensor® (Eiken Chemical Co., Tokyo, Japan) and FOB Gold® (Sentinel, Milan, Italy). ASCCA = Adenoma and Serrated pathway to Colorectal Cancer. Individuals with an indication for surveillance colonoscopy are selected and invited to participate. After oral consent the home stool collection kit is sent to the individuals home address. The test results of Cologuard® (Exact Sciences, Madison, WI, USA) and FIT will be compared to the findings described in the colonoscopy and pathology report in order to yield the test performances. This will then feed the ASCCA model for the simulation of different surveillance strategies. The tissue of lesions removed during the surveillance colonoscopy will be used for molecular analysis of progression biomarkers to define high risk adenomas. This alternative, molecular-based intermediate endpoint will impact the test performances and thus the ASCCA modelling. By adding identified risk factors to the obtained test performance data, new sensitivity data will be acquired and used to repeat the model simulations of surveillance strategies

The primary goal of this study named Molecular stool testing for Colorectal CAncer Surveillance (MOCCAS), is to evaluate the performance of the molecular stool test Cologuard® in post-polypectomy, CRC and FCC surveillance. To this end, we will determine the performance characteristics of Cologuard® in the detection of CRC and advanced adenomas in a surveillance setting. The performance of Cologuard® will be compared to two commercially available FITs (OC-sensor (Eiken Chemical Co., Tokyo, Japan) and FOB Gold (Sentinel, Milan, Italy)) and the gold standard colonoscopy. The test performance data will be used as input in the ASCCA (Adenoma and Serrated pathway to Colorectal CAncer) model that was developed using Dutch data [28]. The model will allow predictions of lifetime health effects and costs for a number of surveillance strategies differing in frequency and duration (Fig. 1).

### Secondary objectives

As a secondary objective, we aim to analyse whether the diagnostic markers included in Cologuard® are present in the tissue samples of lesions identified and removed during the surveillance colonoscopy procedure. These data will then be correlated to the Cologuard® results. In addition, these same tissue samples will be analysed for the presence of particular genomic alterations known to be indicative of progression to cancer [29] and the performance of Cologuard® will be determined for detecting such high risk precursor lesions. This will allow refined analysis of the diagnostic performance of these assays. Through modelling, the impact of using a molecular-based definition of high risk adenoma on predicted health effect and burden will be assessed for alternative surveillance strategies. Moreover, we will incorporate previously identified risk factors for the development of advanced neoplasia in the model [30]. To this end participants will be asked to complete a validated online questionnaire including these risk factors [31].

## Methods

### Study design

This is a multi-centre, cross-sectional observational cohort study in the Netherlands that aims to include 4,000 patients. The study has been approved by the Medical Ethical Committee of the Academic Medical Center (AMC), Amsterdam and has been registered in ClinicalTrials.gov (NCT02715141).

### Study population

All individuals that have an indication for a surveillance colonoscopy according to the previous (2001) [15] or current (2013) [15] Dutch guidelines are eligible for this study. Those guidelines include subjects with a history of polypectomy or CRC, as well as subjects under surveillance for familial colorectal cancer (FCC). In order to complete the risk questionnaire and give informed consent, subjects must have sufficient understanding of the Dutch language. Subjects with inflammatory bowel disease (IBD) or genetic cancer syndromes such as Lynch syndrome, familial adenomatous polyposis (FAP), attenuated FAP (AFAP), MUTYH associated polyposis (MAP), serrated polyposis syndrome (SPS) and other polyposis syndromes are excluded from participation. Also, a colonoscopy in the previous 6 months, having undergone a proctocolectomy or a life expectancy of less than three years are exclusion criteria.

### Study algorithm

#### Subject recruitment and sample collection

Subjects will be invited to participate one to two weeks prior to their routine surveillance colonoscopy by a member of the research team. When oral consent is given, a package containing the study information and -instructions, informed consent form, FITs and stool collection kit will be sent to their home address. Simultaneously, an email containing the link to the online questionnaire will be sent, or a hardcopy version of the questionnaire will be added to the package in case email is not available. This validated questionnaire evaluates the risk factors: age, body mass index (BMI), family history for CRC (first degree relatives), regular aspirin or non-steroid anti-inflammatory drug (NSAID) use, current smoking, history of smoking, alcohol intake, total calcium intake, physical activity and postmenopausal hormone replacement therapy [31]. Regular NSAID intake is defined as the use of NSAIDs three or more times a week during the last month. Calcium intake is estimated by questions about food and supplement intake [31].

For stool collection as part of Cologuard®, a dedicated kit is used as provided by Exact Sciences. This kit comprises materials to collect at home stool from one full bowel movement (whole stool sample), in an easy and hygienic way. Subjects are instructed to collect the stool sample, perform FIT-sampling by swiping the test on the faecal surface, and afterwards add stabilisation buffer to the remaining stool sample for DNA preservation. The sealed samples may be stored at room temperature until the colonoscopy appointment. The allowed time frame between collection of the sample and processing in the laboratory is restricted to 72 h.

On arrival at the endoscopy department subjects will hand in the signed informed consent form and the sealed package containing the whole stool- and FIT-samples. Samples from individuals of whom written informed consent is lacking will be excluded from further processing or analysis. The sealed package will be stored at the endoscopy department until transfer to the laboratory by a courier service.

#### Laboratory procedures

On arrival in the laboratory the FITs and the whole stool sample will be handled separately. The FITs used are automated tests (OC-sensor®, Eiken Chemical Co., Tokyo, Japan and FOB Gold®, Sentinel, Milan, Italy) with a quantitative outcome. The FITs will be analysed on the OC –sensor DIANA (Eiken Chemical Co.) and SENTiFIT 270 (Sentinel) according to the manufacturer’s instructions by an experienced technician, who is unaware of the colonoscopy outcomes. The stool sample will be homogenised, aliquoted and stored at −80 °C. The homogenised samples will be shipped in batches under strict conditions to Exact Science Corporation for analysis. As Cologuard® is composed of a molecular assay plus an immunochemical test for haemoglobin detection, an algorithm derived from these two assays will determine the test result. Researchers performing the analyses will be blinded for the colonoscopy results.

The diagnostic results of the FITs and Cologuard® will be compared to the yield of colonoscopy. Colonoscopy is the gold standard, and neither participants nor doctors will be informed about the test results.

Second, tissue samples of lesions removed during the surveillance colonoscopy procedure will be collected from the pathology archives and subjected to further molecular characterisation. Expression of the diagnostic Cologuard® markers will be tested through methylation-specific PCR and mutation analysis in order to determine which polyps are likely to have contributed to the test result. In a separate analysis, DNA copy number changes that are associated with adenoma to carcinoma progression will be assessed for the identification of high risk adenomas [29]. These changes include losses in 8p21-pter, 15q11-q21, 17p12-13, and 18q12-21, and gains in 8q23-qter, 13q14-31, and 20q13. The presence of two or more of the seven aforementioned chromosomal changes defines a high risk adenoma.

#### Clinical procedures

Colonoscopies (with conscious sedation) will be performed or supervised by experienced gastroenterologists. A complete colonoscopy will be defined as intubation of the caecum with identification of the ileocaecal valve or appendiceal orifice. Quality parameters for colonoscopy will be reported [32, 33]. Patients with an incomplete colonoscopy and/or insufficient bowel preparation will be rescheduled for colonoscopy. Patients that undergo the re-colonoscopy at more than 26 weeks (6 months) after the initial surveillance colonoscopy, and thus collection of the whole stool sample, will be excluded for analyses. Only in case of detection of CRC, an incomplete colonoscopy is no reason for exclusion.

Lesions that are resected during surveillance colonoscopy, will be evaluated by pathologists at the participating centres. Adenomas ≥ 10 mm, with high-grade dysplasia and/or villous characteristics (i.e., tubulovillous or villous adenoma) will be classified as advanced adenomas [13–15]. CRC and/or advanced adenomas are considered advanced neoplasia. CRC will be staged according to the AJCC cancer and TNM staging manual [34].

Formalin-fixed paraffin-embedded (FFPE) tissue samples from all the lesions removed during colonoscopy will be stored in the respective pathology departments of the participating centres. These FFPE blocks will be retrieved for molecular analysis through the Dutch national pathology registry (PALGA) [35] and the Dutch National Tissuebank Portal (DNTP) [36], in the context of the Biobanking and BioMolecular resources Research Infrastructure the Netherlands (BBMRI-NL).

#### Data collection

Clinical data will be collected in a database, which is validated to global regulatory standards. Variables that will be assessed include subject age, sex, indication and date of current surveillance colonoscopy, recommended surveillance interval and findings of the previous colonoscopies. For our main study endpoint, i.e., the accuracy of Cologuard® and FIT in detecting advanced neoplasia, endoscopic- and pathologic characteristics of the lesions found during the subsequent surveillance colonoscopy will be collected.

A dedicated system for registering laboratory- and pathology processes will be used to gather information on the test characteristics and results of Cologuard® and FITs.

### Data analysis

#### Accuracy of Cologuard® and FIT

This study will yield estimates for relative sensitivity and specificity of Cologuard® and FITs versus colonoscopy. The analyses will be based on data from all participants who had valid results on Cologuard® and/or FIT and colonoscopy. In case of missing values on outcome variables, the patient will be excluded. Exact binomial confidence intervals will be calculated around relative sensitivity, relative specificity, positive and negative predictive values.

#### Estimation of lesion specific positivity rates required for model-based analyses

For the cost-effectiveness analyses comparing multiple surveillance strategies, the ASCCA model (Adenoma and Serrated pathway to Colorectal Cancer) will be used. The ASCCA model describes the development of colorectal cancer from adenomatous and serrated precursor lesions. As input, the ASCCA model requires test positivity rates per lesion in each of the model categories of no/small/medium/large adenomas and serrated lesions. These cannot be directly taken from the cross-sectional diagnostic study; the study yields estimates for *relative* sensitivity and specificity of Cologuard® and FITs versus colonoscopy, because colonoscopy is treated as the gold standard. Therefore, we apply a process called calibration. In essence, test-specific positivity rates per lesion are drawn randomly from a wide range of probable values. Subsequently, the model is used to simulate the present cross-sectional surveillance study using these randomly drawn positivity rates. Predictions for the number of positive test results within individuals with small/medium/large adenomas and serrated lesions on colonoscopy are compared to the observed data for Cologuard® and FITs. Sets of positivity rates that reproduce the observed data are kept, whereas estimates that produce predictions that diverge from the data are discarded. For this calibration process, we will run ~10 K+ simulations each involving ~100 K+ individuals (the actual number will depend on stopping criteria for achieving less than a pre-specified level of statistical uncertainty around the predictions). Statistical methods to assess goodness-of-fit will be used to identify the best-fitting sets of positivity rates.

#### Modelling alternative surveillance strategies

The surveillance schedule from the Dutch guidelines ‘Colonoscopy Surveillance’ (2013) [15] will be implemented in the ASCCA model. Subsequently, alternative surveillance strategies will be implemented using the estimates for lesion-specific positivity rates as described in the paragraph above. Different frequencies of molecular testing with Cologuard® and FIT (every 1, 1.5 or 2 years) and rules for referral back to the screening population (after 3, 4, or 5 consecutive negative tests) will be evaluated.

Each model evaluation will involve simulation of 1,000,000 individuals, which represents a ‘virtual’ sample of the Dutch surveillance population. Predicted outcomes will include cancer incidence and mortality, resource utilisation (including number of surveillance tests and demand for colonoscopies) and cost-effectiveness. The evaluation will be accompanied by extensive sensitivity analyses in which, among others, the impact of changes in adenoma and serrated lesion incidence in the surveillance population, test positivity rates in each of the size categories and costs of molecular testing will be evaluated.

#### Modelling the impact of molecularly defined high risk adenomas

In the current version of the model, only advanced adenomas have the possibility to progress to CRC. Advanced adenomas in the model are defined on the basis size and histology. To evaluate the impact of an alternative, molecular-based intermediate endpoint, we will extend the ASCCA model such that it includes the most relevant progression biomarkers [29]. That is, on the basis of these progression biomarkers, the category ‘advanced adenoma’ will be replaced by ‘molecularly high risk adenoma’.

Transition probabilities from the different adenoma health states to a high risk adenoma will be derived by the automatic calibration procedure as described above, such that the molecular version of the ASCCA model also correctly reproduces Dutch age- and sex-specific adenoma prevalence and serrated polyp prevalence, the observed proportion of molecularly high risk adenomas within the subgroups of small/medium/large adenomas, and Dutch CRC incidence and mortality. The result will be the first CRC surveillance model that includes the molecular biology of CRC development.

The model will then be used to repeat the described simulation analyses of alternative surveillance strategies. The hypothesis is that the predictions for health benefits and cost-effectiveness of molecular stool test-based surveillance are underestimated, because the current classification ignores the ability of Cologuard® to detect specifically those adenomas at high risk of progression.

#### Modelling the impact of a risk-based questionnaire

We will build a multivariable logistic regression model by adding all risk factors from the questionnaire and the obtained accuracy data from Cologuard® and FITs, using advanced neoplasia as the dependent variable. Missing data in the questionnaires will be handled by multiple imputations. The newly obtained sensitivity data will be used to repeat the model simulations of surveillance strategies.

#### Sample size calculation

Based on previous reports [27, 37], we assume for the power analysis that Cologuard® has a sensitivity of 50% for advanced adenomas in individuals under surveillance. To obtain an accuracy of 5% around the sensitivity estimate (SE 2.5%, 95% confidence interval of width 10%), a total of 400 individuals with advanced neoplasia are needed. In a recent Dutch surveillance study an advanced adenoma was found in one per ten individuals [22]. Based on this ratio, we will include 4,000 individuals in the current study. This will allow a highly accurate estimate of the specificity of Cologuard® and FIT in this population (estimated width of 95% confidence interval = 2%).

## Discussion

In this cross-sectional observational cohort MOCCAS study, the performance of a multitarget stool DNA test (Cologuard®) and FITs will be assessed as an alternative for surveillance colonoscopy. To this end, the results of the stool based molecular tests and FITs will be compared to the findings of the (routine) colonoscopy in a surveillance population. These data will then be used as input for model-based analyses. By simulating different surveillance strategies varying in testing frequency and rules for referral to the screening population, the model will predict outcomes such as cancer incidence and mortality, resource utilisation and cost-effectiveness.

Multiple modalities are available in the prevention of CRC. Colonoscopy is a one-staged method in which lesions are detected and removed simultaneously. Other methods, such as CT-colonography, faecal immunochemical tests and multitarget stool DNA tests, are two-staged and are used as triage for colonoscopy. The two-staged methods are associated with lower sensitivities, but are generally advantageous in terms of participation rates, risks and costs [27, 28, 38]. When choosing an optimal strategy it should be emphasised that the weight of these factors is different for surveillance compared to screening. Where screening targets the “healthy” population, surveillance is aimed at a narrow high risk group with a higher positivity rate for colonoscopy. Sensitivity of the method is therefore most important. The accuracy of molecular tests might approach colonoscopy in the detection of (high risk) colorectal polyps and –carcinomas when the test is performed more frequently than colonoscopy. Participation in these high risk groups is generally high, as a result of a patient’s awareness of his/her risk.

The optimal model-predicted surveillance strategy, as identified in the current study, will be evaluated in clinical practice through a randomised study. Besides investigating a potential alternative to surveillance colonoscopy, this study will generate new insights in the molecular profiles of precancerous lesions by relating the expression of both diagnostic biomarkers and progression biomarkers to the results of Cologuard®. Moreover, because whole stool samples, as well as FIT samples are collected, a large biobank is established that provides extensive opportunities for future research.

## Abbreviations

AFAP: Attenuated familial adenomatous polyposis
AMC: Academic Medical Center
ASCCA: adenoma and serrated pathway to colorectal cancer
BBMRI-NL: Biobanking and BioMolecular resources Research Infrastructure the Netherlands
BMI: Body mass index
BMP3: Bone morphogenetic protein 3
CRC: Colorectal cancer
DNTP: Dutch National Tissuebank Portal
FAP: Familial adenomatous polyposis
FCC: Familial colorectal cancer
FDA: Food and drug administration
FFPE: Formalin-fixed paraffin-embedded
FIT: Faecal immunochemical test
IBD: Inflammatory bowel disease
KRAS: Kirsten rat sarcoma
MAP: MUTYH associated polyposis
NDRG4: N-myc downregulated gene 4
NSAID: Non-steroid anti-inflammatory drug (NSAID)
PALGA: Pathological Anatomical National Automised Archive (in Dutch: Pathologisch Anatomisch Landelijk Geautomatiseerd Archief)
SE: Sensitivity estimate
SPS: Serrated polyposis syndrome
